# Pretty Cool Beetles: Can Manipulation of Visible and Near-Infrared Sunlight Prevent Overheating?

**DOI:** 10.1093/iob/obac036

**Published:** 2022-08-11

**Authors:** Laura Ospina-Rozo, Jegadesan Subbiah, Ainsley Seago, Devi Stuart-Fox

**Affiliations:** School of Biosciences, University of Melbourne, Building 147, Parkville Victoria 3010, Australia; School of Chemistry, Bio21 Institute - University of Melbourne, 30 Flemington Road, Victoria 3010, Australia; Carnegie Museum of Natural History, 4400 Forbes Ave, Pittsburgh PA 15213, USA; School of Biosciences, University of Melbourne, Building 147, Parkville Victoria 3010, Australia

**Keywords:** Christmas beetles, reflectance, transmittance, size, thermoregulation, structural colour

## Abstract

Passive thermoregulation is an important strategy to prevent overheating in thermally challenging environments. Can the diversity of optical properties found in Christmas beetles (Rutelinae) be an advantage to keep cool? We measured changes in temperature of the elytra of 26 species of Christmas beetles, exclusively due to direct radiation from a solar simulator in visible (VIS: 400–700 nm) and near infrared (NIR: 700–1700 nm) wavebands. Then, we evaluated if the optical properties of elytra could predict their steady state temperature and heating rates, while controlling for size. We found that higher absorptivity increases the heating rate and final steady state of the beetle elytra in a biologically significant range (3 to 5°C). There was substantial variation in the absorptivity of Christmas beetle elytra; and this variation was achieved by different combinations of reflectivity and transmissivity in both VIS and NIR. Size was an important factor predicting the change in temperature of the elytra after 5 min (steady state) but not maximum heating rate. Lastly, we show that the presence of the elytra covering the body of the beetle can reduce heating of the body itself. We propose that beetle elytra can act as a semi-insulating layer to enable passive thermoregulation through high reflectivity of elytra, resulting in low absorptivity of solar radiation. Alternatively, if beetle elytra absorb a high proportion of solar radiation, they may reduce heat transfer from the elytra to the body through behavioral or physiological mechanisms.

## Spanish

La termorregulación pasiva es una estrategia para prevenir el calentamiento excesivo en ambientes con altas temperaturas. ¿Será posible que la diversidad en las propiedades ópticas de los élitros, en los escarabajos de Navidad (Rutelinae), sean una ventaja para evitar que su cuerpo se caliente excesivamente? En este trabajo medimos los cambios de temperatura en los élitros de 26 especies de escarabajos de Navidad, expuestos a radiación en el espectro visible (400–700 nm) e infrarrojo cercano (700–1700 nm), siendo ambas regiones del espectro producidas por un simulador solar. Luego, evaluamos si las propiedades ópticas de los élitros pueden predecir su temperatura final y tasa de calentamiento, controlando por la variable de tamaño. Encontramos que una mayor absorción de luz aumenta las tasas de calentamiento y temperatura final de los escarabajos en un rango significativo a nivel biológico. A su vez, esta absorción varía considerablemente entre especies ya que los élitros estudiados poseen diferentes combinaciones de reflectividad y transmisividad en el visible e infrarrojo cercano. El tamaño fue un predictor importante del cambio en la temperatura final de los élitros (°C después de 5 minutos), pero no en la tasa máxima de calentamiento (°C por segundo). Finalmente, demostramos que los élitros contribuyen a reducir la temperatura corporal cuando se cierran cubriendo la parte dorsal del escarabajo. Proponemos que los élitros altamente reflectivos pueden actuar como una capa semi-aislante, que contribuye a la termorregulación pasiva disminuyendo la absorción de luz. Por otro lado, los escarabajos cuyos élitros absorben una gran proporción de la radiación solar, podrían implementar mecanismos fisiológicos o comportamentales adicionales para reducir la transferencia de calor desde los élitros al cuerpo.

## Introduction

Solar radiation is an important selective pressure shaping the adaptations of organisms. Animals absorb, reflect, or avoid sunlight to maintain their temperatures in a functional range (Norris 1967). In thermally challenging environments, even small variations in temperature (∼<3°C) produced by different optical properties can be biologically relevant to avoid overheating (Smith 2016; Medina et al. 2018; Munro et al. 2019) or resist extreme cold (Zverev et al. 2018). We use the term optical properties to encompass a wide diversity of interactions between natural materials and sunlight, that is, reflectance, absorption, and transmittance in different wavelengths of the solar spectrum. Such passive mechanisms of thermoregulation may be very important for ectotherms since their body temperature depends more directly on the environment (Careau et al. 2015), especially in small animals with rapid rates of heat exchange such as insects (Casey 1992; Amore et al. 2017). However, the role of optical properties in thermoregulation is difficult to quantify since it may be masked by effects of conductive or convective heat, body size, shape, and behavior (Stuart-Fox et al. 2017). In this study, we tested the potential of optical properties as a mechanism for passive thermoregulation in a set of closely related species of beetles with diverse optical properties that occur in challenging thermal environments.

In the absence of an insulating layer, both transmitted and absorbed light increase the temperature of the body due to direct penetration of radiation or conduction. Conversely, when an insulating layer is present, transmittance and reflectance both play important roles. For example, in polar bears (Preciado et al. 2002) and sub-arctic mammals (Walsberg 1991), the increased reflectance of the white fur (which aids in crypsis) is counteracted by maximized transmittance to increase the skin temperature. A similar case of balance between the two properties has been recently described for sunbirds’ feathers (Rogalla et al. 2021). To add another layer of complexity, optical properties may vary with wavelength. Transmittance is influenced by the light scattering properties of the material, both at the cuticle surface and within the integument. In the latter, longer wavelengths penetrate deeper because they are scattered less (Johnsen 2012). Absorption of different wavelengths depends on scattering losses and the chemical composition of the underlying pigments (Johnsen 2012), and in some species the reflectance of near infrared (NIR) wavelengths (700–2500 nm) seems to be largely independent of visible light (Stuart-Fox et al. 2017). Both ultraviolet visible wavelengths (UV-VIS: 300–700 nm) and NIR wavelengths (NIR: 700–2500 nm) are important for radiative heat gain because each waveband accounts for approximately half of the radiant energy in direct sunlight (Stuart-Fox et al. 2017). Studying NIR is particularly relevant since these wavelengths are not likely to be constrained by selection for communication or camouflage (Stuart-Fox et al. 2017). In consequence, different combinations of reflectance, absorption, and transmittance in a wide spectrum should be considered in thermoregulation experiments.

There are very few studies that address the effect of transmittance on heat load in insects because as insulation decreases, the effect of reflectance should increase (Dawson et al. 2014), and it is assumed that insects have comparatively little insulation. Beetles offer a curious case: although they lack a full-body insulation system like birds’ feathers or cetaceans’ blubber, their elytra (modified first pair of wings) fold on top of their secondary wings leaving an air gap between their outermost layer and the body, which may act as a buffer for heat transfer (Bolwig 1957; Heinrich 1993). Some beetles also possess abundant hair-like setae, but these are believed to be more closely tied to defensive mimicry than to thermoregulation (e.g., Glaphyridae: Lichnanthe, Carlson 1980). In most beetles, the elytra are rarely opened outside of flight, and as a consequence they represent the largest area of the beetle exposed to sunlight (unlike butterfly wings in Tsai et al. 2020) so their optical properties may play an important role in passive thermoregulation. Although several studies have explored passive thermoregulation in beetles (Willmer and Unwin 1981; Schultz and Hadley 1987; Turner and Lombard 1990; Carrascal et al. 2017; Zverev et al. 2018), very few have isolated the role of NIR properties (Cuesta and Lobo 2019; Wang et al. 2021) and most of them have been focused on reflectance (With some exceptions: Alves et al. 2018; Cuesta and Lobo 2019). Studying transmittance is important because it can play an additional role in passive thermoregulation for beetles: elytra with high reflectance can shield the body from radiation, but elytra with high absorption and low transmittance may trap the excess of solar energy and redirect it away from the body. Thus, some questions remain: how much variation occurs in both visible and NIR light reflectance/transmittance between species? What is the relative importance of transmittance and reflectance for passive thermoregulation by the elytra? How do the effects of optical properties on heating rates scale with body size?

Christmas beetles (Scarabeidae: Rutelinae) are a good model to address questions of passive thermoregulation because they live in environments in which overheating due to radiation is a significant problem. In Australia, the common name “Christmas beetle” is applied to several genera of Rutelinae scarabs (some genus of the subfamily Melolonthinae, and in Tasmania, lucanid beetles in the genus *Lamprima*) and refers to the fact that the adults of these colorful insects emerge and are active only during the warm Christmas season, that is, December through January (Hangay and Zborowski 2010). During the Australian summer, the conditions can be very challenging: low humidity, high radiation, and temperatures between 26°C and ∼40°C (CSIRO Bureau of Meteorology 2014). The adults are active during the day and are usually found on the top branches of eucalyptus trees in forests and woodlands (Britton 2020). Similar to most beetles, they move slowly and fly only occasionally (Heinrich 1993), which means they spend much of their life time exposed to solar radiation. In addition, the group has highly diverse optical effects including a wide range of NIR properties, iridescent, pearlescent, and metallic appearances (Ospina-Rozo et al. 2022). The optical effects of Christmas beetle elytra are not notably sexually dimorphic and thus presumed not to be subject to sexual selection.

Here, we studied the potential of the optical properties of the beetle elytra in facilitating passive thermoregulation. We conducted high precision experiments to measure small changes in temperature exclusively due to direct radiation from a solar simulator in 56 Christmas beetle specimens from 28 species in 9 genera (Table S1). These taxa were selected because they represent the wide diversity in optical effects in this group. We tested whether the optical properties (absorption, reflectance, and transmittance) of the beetle elytra predict heating (heating rate and total increase in temperature after a given interval of time) in different spectral bands (visible 400–700 nm, NIR 700–1700 nm, and combined 400–1700 nm), while accounting for the effect of size. In addition, we explored correlations between optical properties in different spectral bands. Finally, we quantified the effect of the elytra on the heating rate of the body for one species, *Anoplognathus chloropyrus*. According to the fundamental laws of thermodynamics optical properties are inevitably correlated with heating in a controlled set up (minimal convection or conduction), but from the biological point of view, it is critical to examine if the interspecific variation produces ecologically relevant differences in heating. It is also important to evaluate if the contributions of visible and near-infrared reflectivity vary between species given the high diversity of optical properties of Christmas beetles. We discuss how the interactions between the three optical properties of absorption, reflectance, and transmittance influence passive thermoregulation by beetle elytra.

## Methods

### Museum specimens

We studied 56 Christmas beetles representing 28 species obtained from the Australian National Insect Collection (ANIC) (Table S1). The elytra of the selected taxa have relatively smooth, waxy surfaces without scales or hair. We took photographs of one specimen of each species with a Nikon D7200 DSLR camera and a scale to measure the length of the beetle (in cm). This measurement is a good proxy for elytral area and body size in this group of beetles since their shape is very conserved. Our study consisted of two sets of experiments. In the first, we measured the heating of a single elytron removed from each museum specimen (*n* = 56 individuals, 28 species). In the second experiment, we examined how elytral heating rate corresponds to body temperature in one species, *A. chloropyrus* (*n* = 11 individuals) for which we compared the heating of the body (inserted thermocouple, see below) with and without the elytra. Individuals of *A. chloropyrus* were collected in Orange—New South Wales (33.3234$^\circ $S, 149.0828 $^\circ $E. Collection permit number, FO25000127) in December 2019, and kept in captivity, with heating rate measured soon after their natural death. We used recently dead beetles to avoid error in our measurements due to movement or misplacement of thermocouples, ensure that our experiments excluded additional physiological mechanisms for heat transfer apart from direct radiation, and avoid the infliction of non-trivial discomfort to living beetles.

### Optical properties

To measure the sample reflectance, we obtained hemispherical reflectance spectra of the beetle elytra while still attached to the beetle body with an integrating sphere containing an inbuilt tungsten-halogen light source (400–2100 nm; ISP-REF; Ocean Optics Inc., Dunedin, FL, USA) and 4 mm diameter sampling port (Fig. S1). We did not measure UV due to limitations of the inbuilt light source within the integrating sphere, but this should have minimal effects on conclusions because ultraviolet wavelengths contribute <5% of the energy in solar radiation and Christmas beetles have low reflectance below 300 nm (Ospina-Rozo et al. 2022). The integrating sphere was connected to two spectrophotometers (Ocean Optics Inc., Dunedin, FL, USA), a USB 2000+ (400–1000 nm) and NIRQuest (1000–2100 nm) via a bifurcated optic fibre (Fig. S1). Measurements were recorded using the software OceanView 1.6.7. and calibrated against a diffuse 99% reflectance spectralon standard (Labsphere, North Sutton, NH, USA).

To measure transmittance/direct transmission (Johnsen 2012) we used a standard set up in which the spectrometers and light source are carefully aligned to send a parallel beam of light through the sample and collect, on the opposite side, the portion of the beam that was not absorbed or scattered (Fig. S1). We detached the elytra from the body of the beetle and placed one elytron between the light source and spectrometers. We used two light sources to include the UV-Visible range (PX-2 pulsed Xenon light) and the visible-NIR range (HL-2000 tungsten halogen light), combined via bifurcated optical fibres to illuminate the sample from the dorsal side of the elytra. We used the same two spectrometers connected to a bifurcated optical fibre to capture the light on the other side of the sample. Measurements were recorded in the same software and calibrated against the 100% reference (parallel beam of light without the sample).

Next, we calculated the total percentage of light transmitted or reflected, accounting for the spectral power distribution of the light source. This percentage is termed reflectivity or transmissivity, respectively (definitions and formulae in Table 1). We calculated reflectivity and transmissivity for the different spectral bands: visible (VIS, 400–700 nm), NIR (NIR, 700–1700 nm), and total (Total, 400–1700 nm). The visible spectral band includes 43.1% solar energy, while the NIR band up until 1700 nm accounts for 46.9%. Thus, the spectral range considered in our experiment accounts for ∼90% of solar energy and the total light incident over the samples is similar to that expected under the sun for each of the studied spectral bands (Fig. S2). In addition, the sum of the transmittivity and reflectivity is equivalent to all the light that is not absorbed by the material (Table 1); therefore, we calculated the percentage of absorptivity for the different spectral bands.

**Table 1:** Optical properties. This table contains the definitions (Johnsen 2012) of the optical properties we studied and the equations to calculate the optical properties accounting for the spectral power distribution of the light source and filters used (Smith et al. 2016).

Optical property	Symbol [Units]	Definition	How it was obtained
Irradiance	*I* [W/m^2^]	The number of photons emitted by a source and received by a surface per unit of area	From the manufacturer of the solar simulator
Reflectance	*E* [%]	Radiance of an illuminated object normalized by the radiance of a standard illuminated by the same light	Measured with an integrating sphere from the elytron
Reflectivity	*R* [%]	Fraction of the incident radiation that is reflected by an object	$R = \frac{{\mathop \smallint \nolimits_i^n I\ ( \lambda )\ F\ ( \lambda )\ E\ ( \lambda )\ d\lambda }}{{\mathop \smallint \nolimits_i^n \ I\ ( \lambda )\ F\ ( \lambda )\ d\lambda }}$
Transmittance	Sample = ${\rm{S}}$ [%]Filters = *F* [%]	Amount of light passing through an object normalized by the total amount of light shone on one of the surfaces of that object	Measured with a spectrometer from the elytron and the filters of the set up
Transmissivity	*T* [%]	Fraction of the incident radiation that is transmitted by an object	$T= \frac{{\mathop \smallint \nolimits_i^n I\ ( \lambda )\ F\ ( \lambda )\ S\ ( \lambda )\ d\lambda }}{{\mathop \smallint \nolimits_i^n \ I\ ( \lambda )\ F\ ( \lambda )\ d\lambda }}$
Absorptivity	*A* [%]	Fraction of the incident radiation that is absorbed by an object (determined by its material and geometry)	$A= 100\ - \ \ ( {T + R} )$

### Heating experiments

As the light source, we used a solar simulator (model number: 91,192, Oriel 104 Class A, with AM 1.5 filter; Newport Corp., Irvine, CA, USA) with energy density of 500 W/m^2^ (0.5 Sun) that resembles the solar power distribution (Fig. S2). We obtained the irradiance spectrum of the solar simulator from the manufacturer for the wavelength range 300–1700 nm (Fig. S2).

**Fig. 1:**
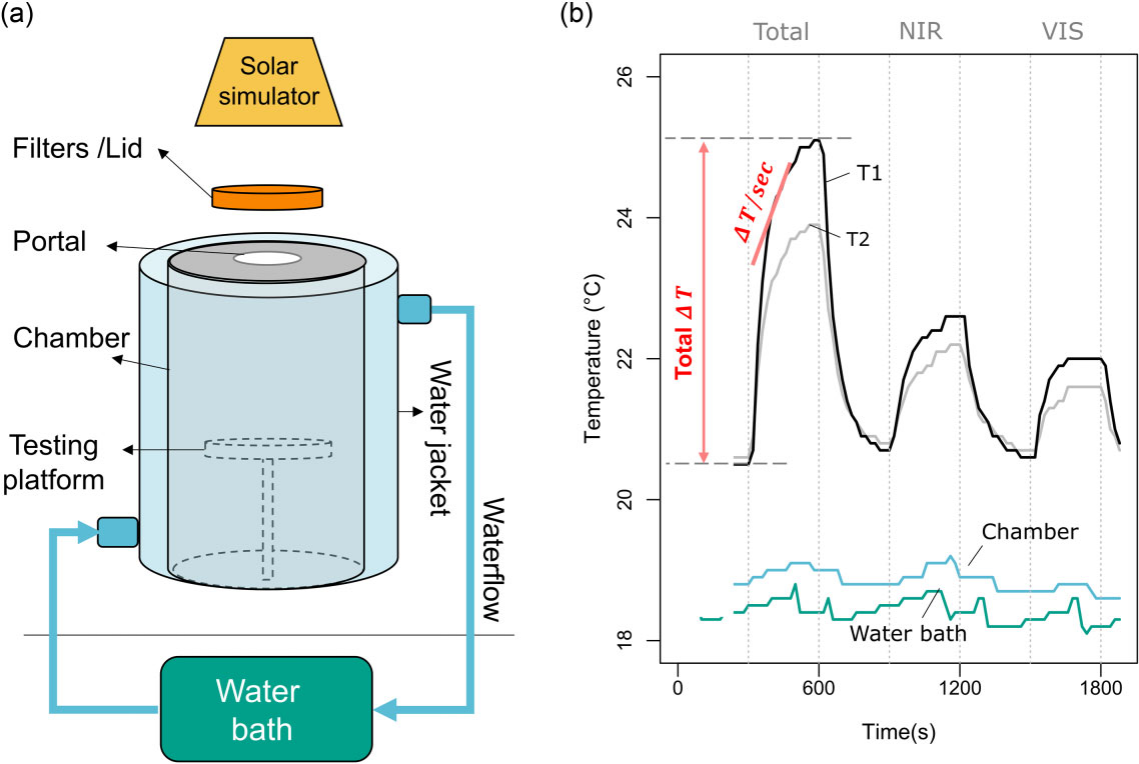
Heating rates experimental set up. (A) We used a solar simulator as the illumination source, filters to test different spectral bands, and a thermal chamber to control the effect of convection or conduction. (B) Example of one cycle of measurements for two samples (T1 and T2). The temperature of the water and the chamber remained constant, while the temperature of the samples increased under different illumination conditions in intervals of 5 min. We used two measurements of heating (red): total change in temperature (ΔT_5) and heating rates (ΔT/sec). The latter was calculated for every pair of points along the heating curve, but only the maximum slope (maxHR) was used in our analyses.

To isolate the effect of the radiation emitted from the solar simulator, we placed the samples in a closed glass thermal chamber directly under the solar simulator, to ensure the sample was illuminated from above while controlling heat exchange by conduction or convection (Fig. 1). We installed a glass water jacket to allow permanent flow of cold water around the chamber. By controlling the temperature of the water bath (measured with a thermocouple) it was possible to keep the temperature of the air inside the chamber constant (measured with a second thermocouple). Inside, samples were placed in the center of a transparent acrylic platform and connected to a thermocouple (ca. 5 mm TP-K01, K type, Center Technology Corp., Taiwan) to record the temperature change throughout the experiment. The thermocouples were connected to a thermometer (Center 521, Center Technology Corp., Taiwan) recording their measurements every 20 s. The glass chamber had one glass portal on top to control the illumination conditions inside the chamber. For full spectrum illumination conditions, the silicon glass portal was uncovered allowing all wavelengths emitted by the solar simulator to be transmitted and illuminate the sample (abbreviated TOTAL). For NIR (700 to 1700) and VIS (400 to 700 nm) illumination conditions, custom optical filter combinations (Edmund Optics) were placed on top of the portal (details in Fig. S2B–C) to only allow transmission of the relevant wavelengths. For periods that required the absence of light (cooling), a lid was placed on top of the portal.

#### Single elytron experiments

In order to study the effect of radiation on a single elytron of different beetle species (*n* = 56), we placed two elytra in the center of the platform inside the chamber and installed one thermocouple touching the ventral surface of each of them (i.e., we conducted the experiment for two elytra at a time). Although it was expected that the area under the glass portal was evenly illuminated, we recorded the side on which the elytra was placed and included it in our analyses to account for any possible effect of this variable. Each trial consisted of alternating 5 min heating and cooling periods in a sequence of: initial cooling, TOTAL, cooling, NIR, cooling, VIS, cooling (Fig. 1B). The initial cooling period was prolonged until the samples reached a stable temperature around 20 degrees. Cooling periods between the different illumination conditions were required to ensure the sample always returned to a similar temperature before starting a new illumination condition.

#### Beetle body experiments

The biological significance of the heating of the elytra depends on the heat transfer between the elytra and the body of the beetle. To confirm whether the elytra play a role in insulating the body, we checked if the presence of the elytra affects body temperature. We placed recently dead individuals of *A. chloropyrus* (*n* = 11) in the center of the chamber and introduced a thermocouple inside its body through the posterior end of the abdomen. We chose this species because it corresponds closely to the average reflectance for our sample in both visible and NIR reflectance: it has a light brown appearance corresponding to a broad band relatively low reflectance and transmittance in the visible spectrum and medium NIR reflectance (Fig. 3B). For these experiments each trial consisted of the same sequence mentioned above allowing the same 5 min of radiation and 10 min of cooling periods because the body has a larger mass and takes longer to dissipate heat. For each individual, we conducted one trial with the elytra closed and one with the elytra open immediately after (Fig. 4A) to compare the heating of the same mass with and without the elytra.

### Heating estimates

We used two different parameters to quantify heating: the total change in temperature after 5 min of illumination ($\Delta {T}_5$, ˚C/5 min) and the maximum heating rate (maxHR = $\Delta T/sec$, ˚C/sec). The latter was calculated as the maximum slope between two adjacent points of the heating curve divided by 20 s (thermometer data collection interval) (Fig. 1B). These two parameters are expected to be affected differently by the size of the object: large objects are expected to have greater steady state temperatures but slower heating rates than small objects. It is reasonable to assume that $\Delta {T}_5$ is indicative of the steady state temperature since most of our samples started to plateau after 5 min under any of the three illumination conditions.

### Statistical analysis

All statistical analyses were conducted in R 4.1.2 (R Core Team 2021). We conducted a repeatability test on repetitions of the $\Delta {T}_5$ and maxHR measurement for the elytra of *A. aureus* (*n* = 7), *P. olivaceous* (*n* = 7), *A. prasinus* (*n* = 9), *C. rayneri* (*n* = 7), *R. manicatus* (*n* = 6), *and A. viriditarsis* (*n* = 8), for each of the spectral bands (TOTAL, NIR, and VIS). We used a bootstrapping tool for gaussian data and calculated the percentage of variance explained by the groups and the *P*-value based on a likelihood ratio test (rpt, R package rptR; (Stoffel et al. 2017).

#### Single elytron experiments

We used linear models to test if elytra absorptivity (*n* = 56 beetle elytra) predicted $\Delta {T}_5$ and maxHR under different illumination conditions (TOTAL, NIR, and VIS). We included the size of the elytra as a fixed effect, since it was expected to influence heating rates due to differences in mass and area. We did not include interaction terms in our models because there was no *a priori* reason to expect such interaction: in our set up, the thermodynamical law of conservation of energy can be applied since any increase in temperature (molecular kinetic energy) is a consequence of the absorption of radiative energy (Shankar 2014). Increased absorption of light can only be produced by increased absorptivity per unit of area (as measured here) or increased total area exposed (here size). Thus, as long as the illumination is even across the surface (not a laser or a focused beam), the heating of the sample depends on the additive effects of absorptivity and size, while synergistic or antagonistic effects (interactions) are not expected. Importantly, the absorptivity of a material depends on its refractive index, the optical properties of its pigments and the geometry of its structures. The latter can be altered by the size of the object, but this is still an additive effect since ultimately it will affect the total amount of light absorbed. After fitting the linear models, we checked the distribution of the residuals to confirm the assumption of normality with mean = 0 and constant variance. We used Pearson's correlation to explore correlations between transmissivity and reflectivity, between different spectral bands, and between elytra optical properties and size. We did not use phylogenetic correction because our experiment is designed to explore the biophysical relationship between heating rates and optical properties of the materials, which is independent of any evolutionary associations. That is, heating rate is a physical consequence of reflectivity, rather than a trait that can evolve independently from reflectivity (or evolve together with reflectivity in response to similar selective pressures); therefore, it is not appropriate to test for correlated evolution of reflectivity and heating rate using phylogenetic comparative methods. Additionally, the optical properties of Christmas beetles’ elytra vary greatly among congeneric species and show no evidence of clustering by genus. (Ospina-Rozo et al. 2022).

#### Beetle body experiments

We tested if there is a greater $\Delta {T}_5$ in the beetle body when the elytra are not covering the body by using a non-parametric paired analysis. For each individual, we calculated the difference in $\Delta {T}_5$ when its elytra were open and when they were closed $({D}_{\Delta T}$) (equation in Fig. 4A). Then, we used bootstrapping to estimate a 95% confidence interval around the mean of the difference (${D}_{\Delta T}$) in a group of 11 recently dead beetles. A negative interval not including 0 indicates that the change in temperature of the beetle body is greater when the elytra are not covering it. We repeated this procedure for each of the three spectral bands.

## Results

### Repeatability of the heating measurements

Repeatability was high for the measurements of $\Delta {T}_5$ in the three illumination conditions: TOTAL $R\ = \ 0.91$, NIR $R\ = \ 0.79$, and VIS $R\ = \ 0.78\ $(Table S2). This result, along with the small standard error in our estimates in the linear models (Table 2) showed that our set up is reliable to detect very small changes in temperature due to radiative heat in the range 0.7 to 5.3°C/5 min. Repeatability of maxHR was considerably lower: TOTAL $R\ = \ 0.42$, NIR $R\ = \ 0.64,$ and VIS $R\ = \ 0.22$

(Table S2). This result is expected since the calculation of maxHR was obtained as the derivative of the heating curves at the very fine scale of 0.01 and 0.105°C/s. However, our analysis also showed that despite the low values of repeatability for this calculated value, the differences between species are still detectable in the data set (*P* value for TOTAL and NIR < 0.01 and for VIS a marginal 0.059; raw results for both heating measurements in Fig. S3). Due to low repeatability of the maximum heating rate, our inferences are based more strongly on the final temperature.

**Table 2:** Results of the linear models. Results of 6 models testing how heating ($\Delta {T}_5$ and maxHR) is predicted by absorptivity and size (elytra length) in each spectral range. *P*-values < 0.05 are highlighted.

Response variable	Spectral range	Overall R^2^ (%)	Parameter	Partial R^2^ (%)	Estimate	Std error	*P-value*
$\Delta {T}_5$ (°C/5 min)	Total	53.65	Intercept	NA	2.024	0.334	**< 0.001**
			Absorptivity	47.08	0.029	0.004	**< 0.001**
			Size	4.67	0.274	0.124	**< 0.05**
	NIR	32.17	Intercept	NA	1.13	0.192	**< 0.001**
			Absorptivity	30.57	0.010	0.002	**< 0.001**
			Size	0.97	0.069	0.083	0.412
	VIS	43.43	Intercept	NA	0.201	0.235	0.396
			Absorptivity	14.08	0.008	0.002	**< 0.05**
			Size	20.76	0.222	0.053	**< 0.001**
*maxHR* (max. $\Delta $°C/sec)	Total	29.91	Intercept	NA	0.0322	0.0125	**< 0.05**
			Absorptivity	22.53	0.0006	0.0002	**< 0.001**
			Size	1.1	0.0036	0.0046	0.432
	NIR	30.33	Intercept	NA	0.0263	0.0043	**< 0.001**
			Absorptivity	22.21	0.0002	0.0001	**< 0.001**
			Size	4.28	−0.0035	0.0019	0.067
	VIS	24.27	Intercept	NA	0.0026	0.0097	0.788
			Absorptivity	11.62	0.0003	0.0001	**< 0.05**
			Size	0.34	0.0007	0.0022	0.763

*Note:* The three spectral bands were TOTAL (400–1700 nm), NIR (700–1700 nm) and VIS (400–700 nm). Units of the estimates and standard errors are specified in the response variable column. The reported estimates already account for the effect of the side of the platform in which the samples were placed. The difference between the overall R^2^ and the sum of the partial R^2^ corresponds to the variance (%) explained by the side. Its effect was significant (p < 0.05) only for VIS, and *maxHR* TOT and VIS.

### Effect of optical properties on the heating of single elytra

**Fig. 2:**
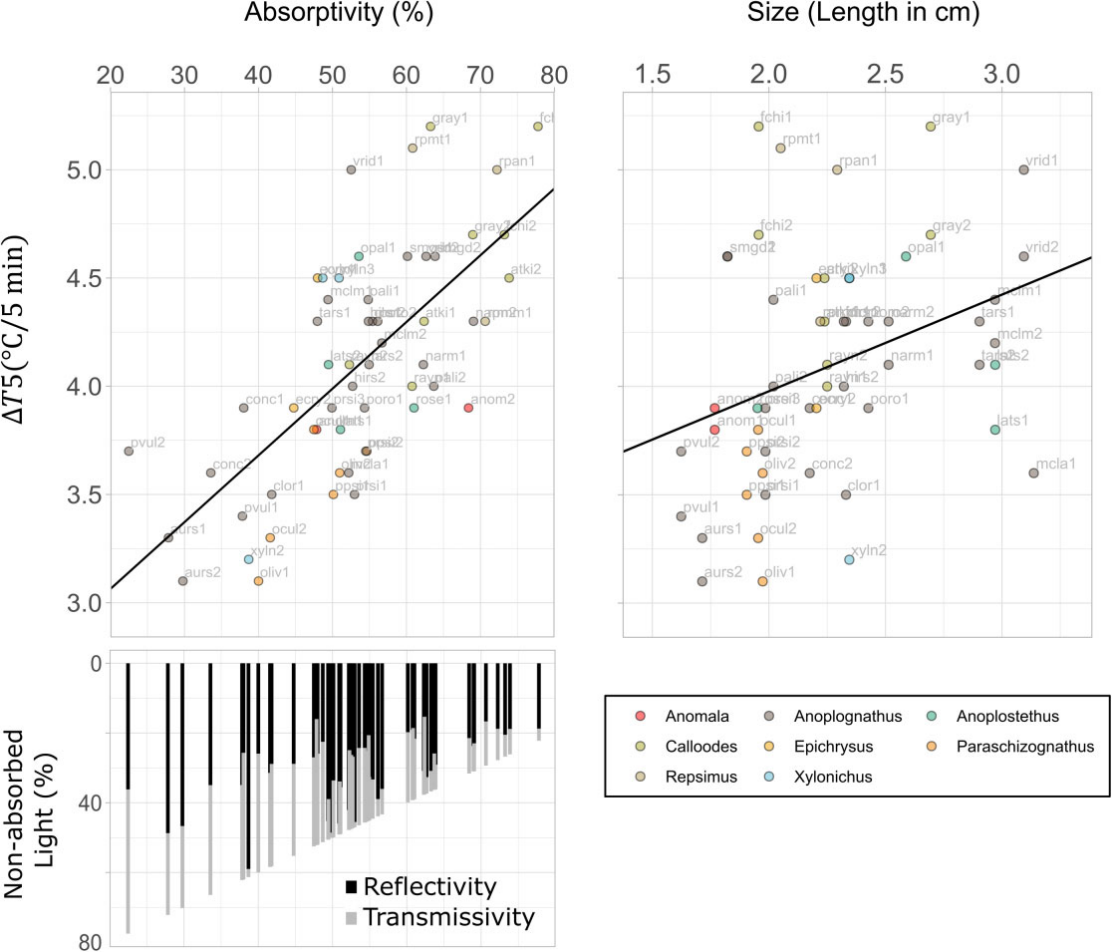
Example of the effect of absorptivity and the size on heating of single elytra. Higher percentages of absorptivity and larger elytra predict a greater increase in temperature due to radiative heat gain in the Total spectral band from 400 to 1700 nm (top panels). Variation in the absorptivity of Christmas beetle elytra is produced by different combinations of transmissivity and reflectivity (bottom left). We sampled 28 species from 8 genera of Christmas beetles (bottom right).

We found that higher absorptivity significantly increased both $\Delta {T}_5$ and maxHR in all three illumination conditions (Fig. 2; Table 2) after controlling for size. Absorptivity explained 14–47% of the variation in $\Delta {T}_5$, while reflectivity by itself explained only 5 to 21%. Absorptivity explained 11–23% of the variation in maxHR, while reflectivity by itself explains 8–21% (partial ${R}^2$ in Table S3). Transmissivity by itself was not a significant predictor of the heating measurements (Table S4). Our results show that combining transmissivity and reflectivity to estimate absorptivity improved the power of our models to predict $\Delta {T}_5$ and maxHR.

### Correlations between the optical properties of the elytra

**Fig. 3:**
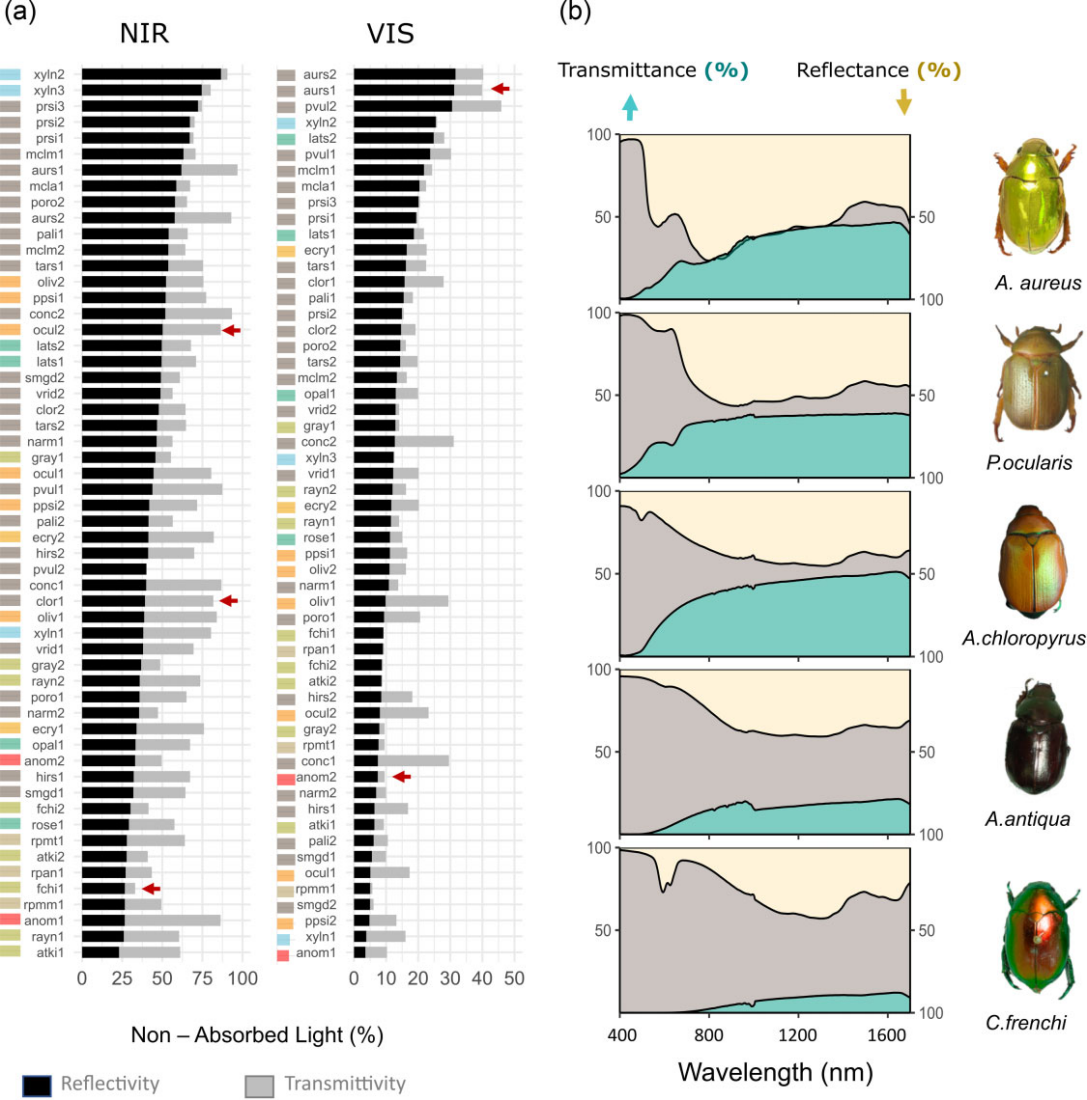
Variation in optical properties of Christmas beetle elytra in NIR and VIS spectral bands. (A) In general, elytra absorb a higher percentage of VIS wavelengths than NIR wavelengths (note difference in x axis). Colours correspond to the genus (consistent with Fig. 2, bottom right). (B). Raw spectra of some of the studied species (also marked in panel A with red arrows). Reflectance is always at the top with an inverted axis. Grey represents the light absorbed by the elytra.

A negative correlation between reflectivity and transmissivity was expected since the light that is reflected cannot be transmitted by the elytra. Interestingly, we only found a significant correlation between reflectivity and transmissivity in TOTAL (−32%, $P < 0.05$) and NIR (−47%, $P < 0.01$) spectral bands, but not in VIS ($P\ = \ 0.84$; Table 3). We observed considerable variation in the combinations of transmissivity and reflectivity in the two spectral bands studied (NIR and VIS; Fig. 3). In addition, we observed that the percentage of light absorbed by the beetle elytra was much higher in VIS than in NIR (Fig. 3). Reflectivity in VIS ranged between 5 to 30%, while in NIR it ranged from 25 to 90%, and on average transmissivity was also higher in NIR than in VIS. We evaluated the extent to which the optical properties in the two different spectral bands (VIS and NIR) may be correlated for this group of beetles (Table 3). A correlation between NIR and VIS light manipulation is expected, since light manipulation depends on structures or compounds tuned to specific wave bands; however, the strength of this correlation may vary in different groups of animals. We found a strong correlation between NIR and VIS transmissivity (Pearson's coefficient R = 0.75) and absorptivity (Pearson's coefficient R = 0.78). However, the correlation between NIR and VIS reflectivity was only R = 0.58. This result indicates that for some species high reflectivity in NIR is accompanied by low reflectivity in VIS or vice versa.

**Table 3:** Correlations between parameters. Pearson's correlation coefficient, confidence intervals and *P*-values are given for each correlation. *P*-values < 0.05 are highlighted.

Correlations involving:	Parameters	Spectral range	Pearson's R	95% confidence interval	*P-value*
Optical properties	Transmissivity x Reflectivity	Total	−0.325	−0.542, −0.068	**0.014**
		NIR	−0.477	−0.654, −0.239	**< 0.001**
		VIS	0.027	−0.289, +0.237	0.842
Spectral Bands NIR—VIS	NIR—VIS Transmissivity	–	0.750	+0.607, +0.846	**< 0.001**
	NIR—VIS Reflectivity	–	0.585	+0.381, +0.735	**< 0.001**
	NIR—VIS Absorptivity	–	0.782	+0.653, +0.866	**< 0.001**
Size and optical properties	Size x Reflectivity	Total	0.124	−0.143, +0.375	0.361
		NIR	0.130	−0.137, +0.379	0.339
		VIS	0.062	−0.204, +0.319	0.651
	Size x Transmissivity	Total	−0.343	−0.556, −0.088	**0.009**
		NIR	−0.364	−0.572, −0.112	**0.006**
		VIS	−0.204	−0.443, +0.062	0.131
	Size x Absorptivity	Total	0.197	−0.068, +0.438	0.144
		NIR	0.247	−0.017, +0.479	0.066
		VIS	0.076	−0.191, +0.332	0.578

### Effect of size on the heating rate of single elytra

Larger elytra had a greater $\Delta {T}_5$ in TOTAL (example in Fig. 2) and VIS but not under NIR illumination (Table 2). We did not find evidence of an effect of size on the maxHR in any of the three illumination conditions (Table 2). These results show that the variation in size in this group is only a significant predictor of variation in the final temperature after 5 min, but not in heating rates. Size was not correlated with reflectivity nor absorptivity, but it was negatively correlated with transmissivity in TOTAL (−34%) and NIR (−36%) spectral bands (Table 3). Thus, only the transmissivity in NIR scaled with the size of elytra, while the other optical properties were independent of elytra size, for the modest degree of size variation in our dataset.

### Effect of the elytra in the heating rate of the full body

**Fig. 4:**
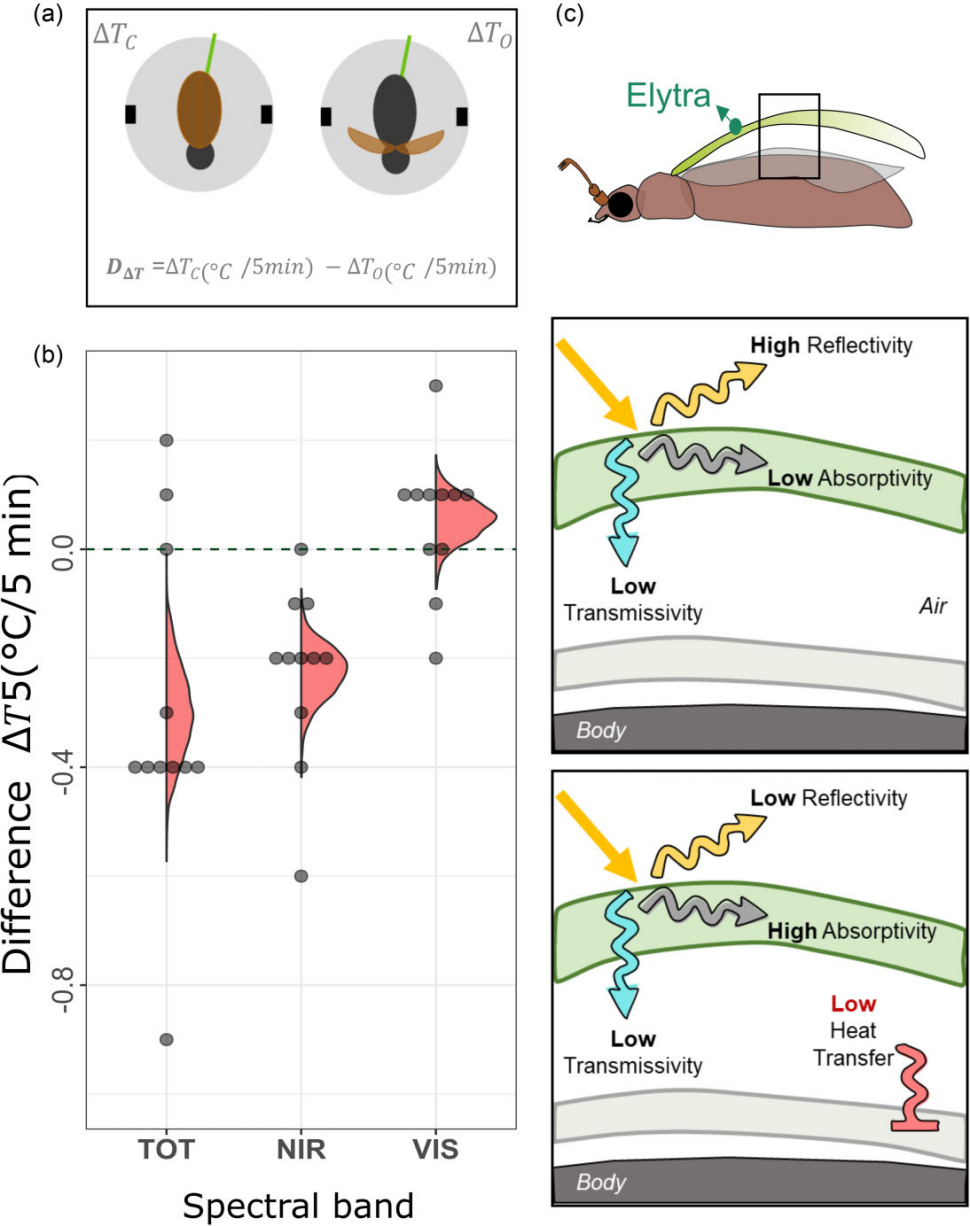
Effect of the elytra on heating of the body of *A. chloropyrus*. (A) Graphical representation of our test statistic for the non-parametric paired test. (B) *D*_Δ*T*_ for each spectral band. A negative value indicates that the increase in temperature of the beetle body is greater when the elytra are not covering it. The half violin plots represent the results of the bootstrap loop. *D*_Δ*T*_ is significantly different to 0 only under full and NIR illumination. (C) Two potential strategies to avoid overheating. Beetles could have elytra with high reflectivity or low transmissivity, both resulting in low heat transfer from the elytra to the body.

The body of *A. chloropyrus* had higher $\Delta {T}_5$ when it was not covered by the elytra, i.e. negative difference in the paired experiment (Fig Fig. 4A) in TOTAL (95% C.I. −0.482, −0.127) and NIR (95% C.I. −0.318, −0.136) illumination, but not in VIS (95% C. I. −0.0182, 0.127) since the 95% C. I. overlapped with 0 (Fig. 4B).

## Discussion

Are pretty beetles also cool beetles? The astonishing diversity in appearances of Christmas beetles is determined by the way their cuticle interacts with light. We evaluated how different optical properties of beetle elytra affect heating due to radiation in different spectral bands: visible (400–700 nm) and NIR (700–1400 nm). We found that absorptivity (the combination of reflectivity and transmissivity) is a better predictor of the temperature and heating rate of Christmas beetle elytra than reflectivity. Lower absorptivity corresponds to lower heating rate (maxHR) and cooler final steady state ($\Delta {T}_5$) of the elytra. Christmas beetles vary greatly in their absorptivity by combining different degrees of reflectivity and transmissivity in both VIS and NIR. Their optical properties are only weakly correlated in NIR and not correlated in VIS, while high reflectivity in NIR is not restricted to species with high reflectivity in VIS (58% correlation). Moreover, relatively small variations in size amongst species can also affect heating. Consequently, different species of Christmas beetles can have elytra with different potential for passive thermoregulation and achieve this by different combinations of optical properties. Since the presence of the elytra covering the body of the beetle can reduce the heating of the body itself, we propose that the interactions of the beetle elytra with light could help some species to keep cool.

Our experiment was able to detect very small changes in temperature due to radiation, but are these changes biologically significant? The final steady state of the elytra $\Delta {T}_5$ ranged from 3 to 5°C, while having the elytra folded on top of the body reduced the $\Delta {T}_5$ of the body of *A. chloropyrus* by 0.2 degrees. These changes may seem smaller than the difference of 5°C between treatments found by Carrascal et al. (2017—Comparison: final temperature after 8 min of natural light illumination ventral vs. dorsal orientation). However, our glass chamber was designed to minimize heating by conduction or convection and our initial temperature was kept at 20°C for all experiments. If these two variables are not controlled, air temperature can rapidly increase due to the heat generated by the light source. Conduction can then contribute to the heating of the sample, inflating the estimated effect of reflectivity. Conversely, our results reflect only the differences caused by radiative heat, which can be a decisive factor for passive thermoregulation in hot environments (Shi et al. 2015).

The differences of 3 to 5°C in of $\Delta {T}_5$ we registered between beetle elytra are within the range of other biologically significant values. For example, a ∼5°C increase in temperature can make the difference between non-active to active status in jewel beetles (Bonsignore and Bellamy 2007) and a decrease in temperature of ∼ 1.5 and 6°C can make the difference between thoracic temperature during and post flying (as an indication of a cooling down process) in burying beetles (Merrick and Smith 2004). In a similar way, relatively small temperature differences could make the difference between remaining at a peak of optimal performance temperature and reaching a critical upper thermal limit. Although the difference in body temperature with and without elytra that we registered (0.2°C) is small, *A. chloropyrus* has moderate reflectivity, and the difference in $\Delta {T}_5$ for species with higher reflectivity is expected to be higher. Thus, the optical properties of the elytra could influence the beetles’ fitness by affecting the amount of time spent feeding or mating rather than sheltering to avoid overheating (Clusella Trullas et al. 2007; Umbers et al. 2013). Further experimental studies are required to quantify the contribution of these optical properties relative to other factors such as metabolic production of heat, body mass, shape, and behavior, to ultimately estimate their effect on the fitness of individuals.

An outstanding question about the relevance of light manipulation for thermoregulation is: what is the correlation between optical properties and to what extent do they vary across different spectral bands in a given taxonomic group (Stuart-Fox et al. 2017)? Previous studies comparing visible and infrared light reflectance and transmittance have had limited capacity to address this question because they focus on a relatively small number of species (e.g., Amore et al. 2017; Cuesta and Lobo 2019–7 and 2 species, respectively). In our study, we evaluated 28 optically diverse, closely related species. We found that, unlike other groups, Christmas beetles can achieve a wide range of combinations of optical properties in different spectral bands. For example, reflectivity and transmissivity are expected to be negatively correlated, but they showed only a weak correlation in NIR and no correlation in visible wavelengths. In a similar way, the optical properties in visible and NIR are expected to be correlated (for example, a highly reflective red patch is expected to also have some reflection in the NIR). Our results supported this prediction, but the correlation between NIR and VIS was notably higher for transmissivity (∼75%) and absorptivity (78%) than reflectivity (58%). This may be explained by their underlying mechanisms. Transmittance and absorption may be primarily determined by cuticle thickness and presence or absence of underlying pigments, whereas reflectance is likely to involve modular and hierarchically ordered nanostructures optimized to interact with different spectral ranges. As a result, reflectance in visible and infrared spectral bands can vary more independently in response to selection for different functions. This has also been observed in other species such as birds (Stuart-Fox et al. 2018), butterflies (Tsai et al. 2020), and chameleons (Teyssier et al. 2015).

Our results suggest that the metallic appearances associated with broadband reflectance (such as gold, silver, and brassy) of some beetles have higher potential for passive thermoregulation than narrow-band structural colors. Metallic colors, produced by multilayer reflectors in the beetle elytra (Thomas et al. 2007; Seago et al. 2009), have traditionally been considered to be unrelated to thermoregulation (Heinrich 1993). However, in our sample, strongly metallic species such as *A. aureus* and *A. parvulus*, have broadband and high overall reflectivity with corresponding low heating, while species with narrow band deep green colors (e.g., *A. smaragdinus, C. atkinsoni*, and *C. rayneri*) have low reflectivity and comparatively high heating due to radiation. Some species such as *A. viriditarsis, A. chlorpyrus*, and *A. porosus* have a sheen of metallic color, but the heating of their elytra is more strongly determined by the total reflectance arising from the underlying brown (melanin) layer than by the top layers producing the metallic sheen. Although in some studies it has been mentioned that “metallic” colors can reach very limited reflectance (Alves et al. 2018), this may be because the term was used to refer to narrow-band iridescent colors. Instead, the nanostructures that produce metallic gold appearances can result in high reflectivity across a broad range of wavelengths (Parker et al. 1998; Feller et al. 2017). Although the biological significance of these highly metallic appearances is still unclear (Thomas et al. 2007; Franklin et al. 2021), it may be premature to exclude a thermal function. Indeed, if high-reflectance optical mechanisms are effective in preventing the insect from overheating, this could be a factor of explaining the apparent concentration of bright metallic-colored insects in the warmer latitudes of the globe.

We demonstrated that it is important to consider NIR wavelengths in studies of radiative heat gain. The optical properties in these wavelengths predict the heating rates and final temperature of both the elytra and the beetle body. Most previous studies of passive thermoregulation in insects have not isolated the effect of visible light from the NIR wavelengths (Umbers et al. 2013). The studies that do consider these different wavebands provide mixed evidence: for tiger beetles, NIR reflectivity seems to be present only in white patches and has a limited role in thermoregulation (Schultz and Hadley 1987), while jewel beetles have high variability in NIR reflectance, which in turn, has a strong effect on the heating rates of their elytra (Wang et al. 2021). Our sample consisted of species with high diversity in NIR reflectivity ranging between 23–86%, and NIR transmissivity ranging from 2–60%. We found that the lowest heating rates and final temperature of the elytra were observed for species with the lowest NIR absorptivity, achieved either by high NIR reflectivity (*X. eucaliptii, A. prasinus, A. aureus*, and *P. olivaceous*) or high NIR transmissivity (*A. concolor* and *P. ocularis*). Therefore, we conclude that manipulating NIR wavelengths can be an effective mechanism to aid in passive thermoregulation for some species of Christmas beetles. The importance of the NIR wavelengths will depend on the biology of the species and the variability in thermal environments and optical properties. Future studies could explore to what extent NIR reflectance and transmittance vary in the different families of beetles and whether this corresponds to specific thermal environments.

In addition to the optical properties, size is also a relevant factor for thermoregulation. Generally, larger organisms are predicted to have a higher steady state temperature, but slower heating rates and vice versa (Porter and Gates 1969; Dzialowski 2005). In our experiment, size predicted $\Delta {T}_5$ but not heating rates. In jewel beetles (length∼ 1.2 to 4 cm), size (effective area) predicted heating rates of isolated elytra under full spectrum illumination (Wang et al. 2021). Despite testing a larger sample size, we found no effect of size on heating rates possibly because Christmas beetles are more homogeneous in size (∼1.6 to 3 cm length) and shape. On the other hand, size significantly determined $\Delta {T}_5$ under VIS, but not under NIR radiation. This may be because absorptivity was higher in VIS than NIR, and a larger area of elytra with high absorption is associated with a higher increase in temperature (Stewart and Dixon 1989), making the effect of size easier to detect. Finally, the negative correlation between size and NIR transmissivity may be because longer wavelengths penetrate deeper in the structures undergoing more scattering/absorption in a thicker, more complex cuticle likely associated with larger elytra (Johnsen 2012; Cuesta and Lobo 2019).

Traditionally, only reflectance is considered in thermoregulatory studies for insects assuming a lack of insulation. Alves et al. (2018) suggested that higher transmittance from the ventral side to the exterior of the elytra could facilitate heat dissipation. Although we found differences in transmittance of light incident on the dorsal and ventral sides of the elytra (Fig. S4), under natural circumstances, light in the solar spectrum would not be transmitted from the ventral side when the elytra are closed. Additionally, optical properties in the solar spectrum are not relevant to heat dissipation from the body to the environment, which is a function of emissivity in the mid- to long wavelength range, that is, 4000 to 50,000 nm (Nussear et al. 2000). A recent study on heat transfer through the iridescent feathers of sunbirds showed that the melanin granules (whose ordered arrangement produces the iridescence) enhance absorption and reduce transmittance of light towards the skin potentially preventing heating up if combined with mechanisms to reduce heat transfer to the skin (Rogalla et al. 2021). Our results showed that combining reflectance with transmittance (i.e., absorption) helps to better predict the heating in this group of beetles in contrast to only considering reflectance. Elytra with high reflectance can reduce heating, but elytra with high absorption could still achieve a similar result if combined with a mechanism to reduce heat transfer towards the body, and this is only possible because of their low transmittance. Such mechanisms can include morphological strategies such as increasing the size of the air layer underneath the elytra (Heinrich 1993), as well as physiological or behavioral strategies to allow rotation of the air such as controlled breathing (Duncan 2021) and periodically opening the elytra (“ventilations” in Bolwig 1957). Thus, two strategies could be effective for passive thermoregulation by the elytra to avoid overheating: Christmas beetles may either use their elytra as a shield against radiative heat gain or employ physiological or behavioral mechanisms to dissipate the radiative heat absorbed by the elytra. Whether both strategies are used by species in thermally challenging environments requires observation of beetles in their natural environment.

## Supplementary Material

obac036_Supplemental_File

## Data Availability

The original data and code for this manuscript are available as an interactive file here: https://lospinarozo.github.io/PrettyCoolBeetlesCodeAndData/ and they can also be found in Dryad in: https://doi.org/10.5061/dryad.7wm37pvx4.
