# Supplementary material for: Pretty Cool Beetles: Can Manipulation of Visible and Near-Infrared Sunlight Prevent Overheating?
Source: Integr Org Biol. 2022 Aug 11;4(1):obac036. doi: 10.1093/iob/obac036 (PMC9470487; doi:10.1093/iob/obac036)
Supplement: obac036_Supplemental_File [file obac036_supplemental_file.docx]

**Pretty cool beetles: can manipulation of visible and near-infrared sunlight prevent overheating?**

Supplementary Materials

**Table S1.** List of species and ANIC reference number of the specimens used in this study.
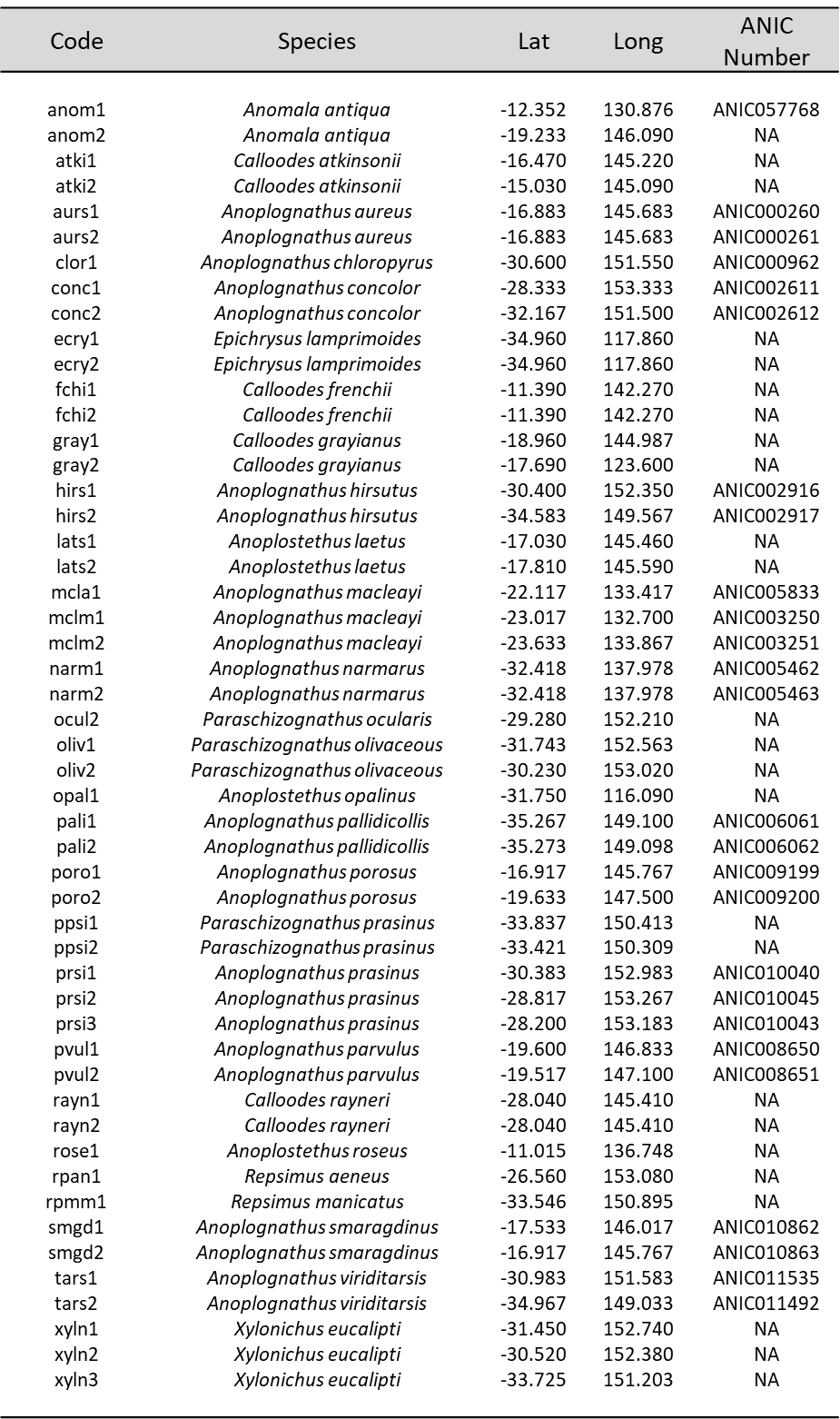


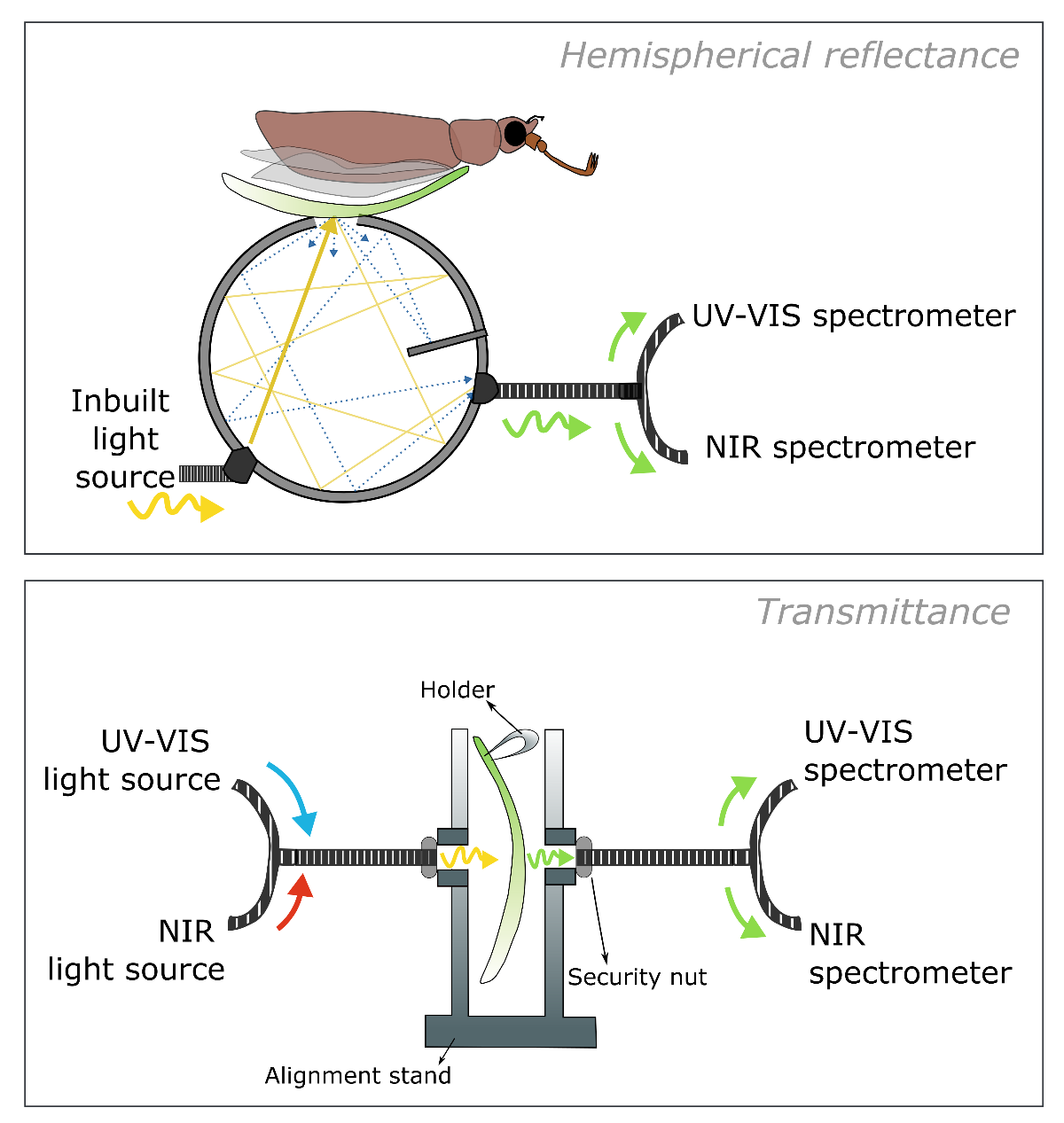


Figure S1. Setup to measure the optical properties. We measured hemispherical reflectance (reflectance integrated across all angles) on the elytra still attached to the beetle body with an integrated sphere. The sample is illuminated with a broadband source (400 – 2100 nm) and the reflected light is collected by a bifurcated fibre that connects to two spectrometers UV-VIS (400-1000 nm) and NIR (1000-2100 nm). We measured transmittance in one elytron from each beetle specimen. To quantify the amount of light that can pass through it is crucial to ensure proper alignment. Thus, we used a metallic stand with two plaques with opposite pinholes. A security nut fixes the light and spectrometer fibre optics to the stand in the optimal position. We carefully suspended the elytron between the two plaques with a custom-made holder.

^
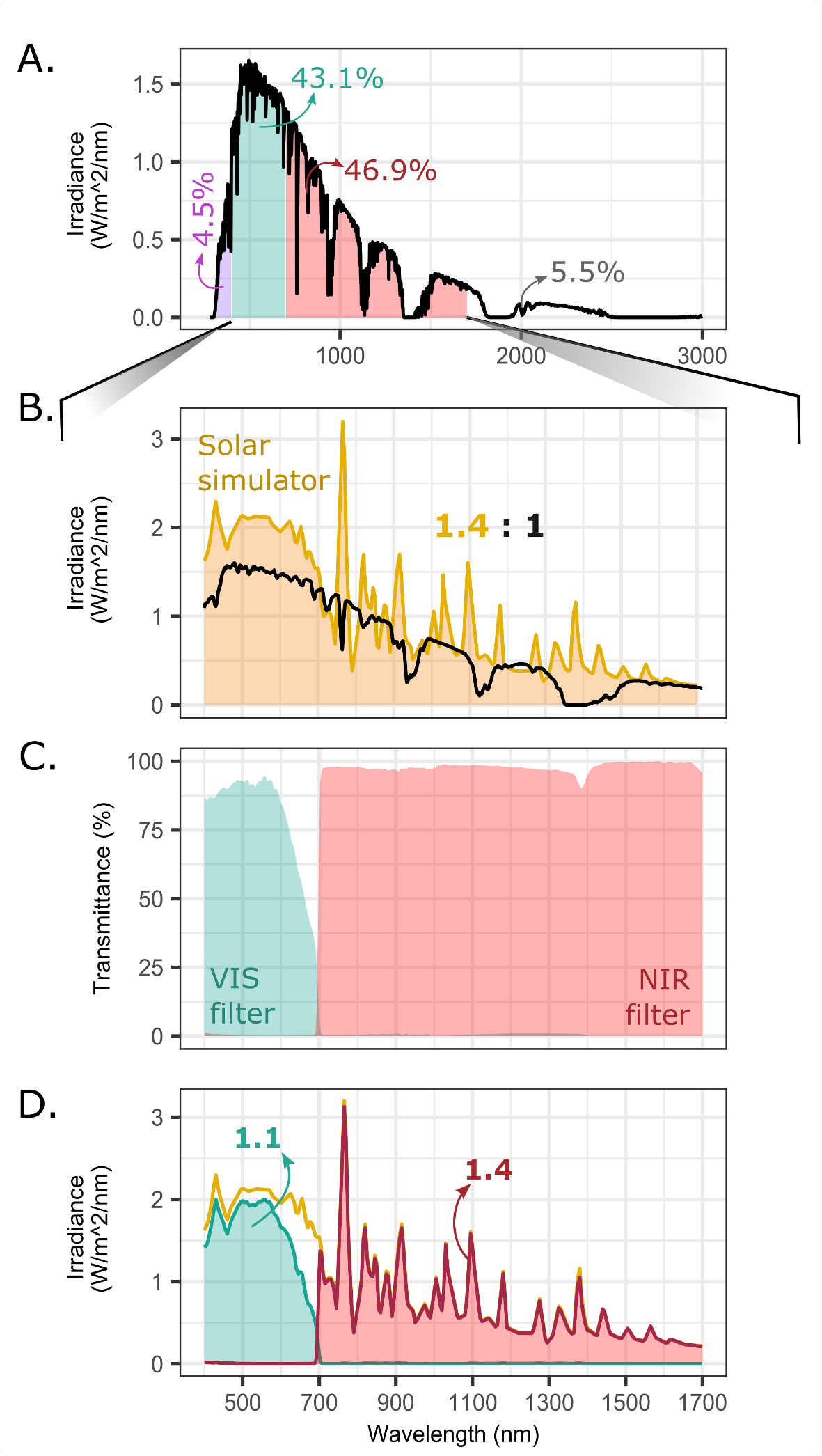
^

**Figure S2. Irradiance spectrum of solar simulator and filters.** A) Irradiance spectrum of the sun and the percentage of energy associated to each spectral band UV, visible and NIR. The UV (wavelengths shorter than 300nm), and in the NIR beyond 1700 nn (not shaded) were not considered in our experiment, but together they only represent 10% of the energy available from the sun. B). Comparison between the spectrum of the solar simulator at 0.5 Sun (shaded area) and the solar (black lines) irradiance (W/m^2^ nm) in the wavelength range relevant for our experiment 400 to 1700 nm. Although the solar simulator has higher irradiance in the VIS region than the sun, the overall shape is similar. The numbers indicate the ratio solar simulator: solar irradiance energy in this wavelength range. C) Transmittance of VIS and NIR filters used in our experiment. These filters consisted in the following combinations of optical filters manufactured by Edmund Optics (Singapore): items #84727 and #49095 [KG-5 heat absorbing glass] were used for the VIS transmitting filter and items #84760 and #64703 were used for the NIR transmitting filter. D) Proportion of the solar simulator irradiance effectively reaching the platform inside the chamber after passing through each of the filters (shaded), in comparison to the original irradiance of the solar simulator (black line).The numbers indicate the ratio of effective incident light compared to the solar irradiance energy for each of the two wavelength ranges visible and NIR. The fact that the ratio is close to 1 in the three intervals used in our experiments (VIS, NIR and total) indicates that amount of light incident over our sample is similar to that expected under the sun.

**Table S2.** Repeatability (R) of heating measurements.

**
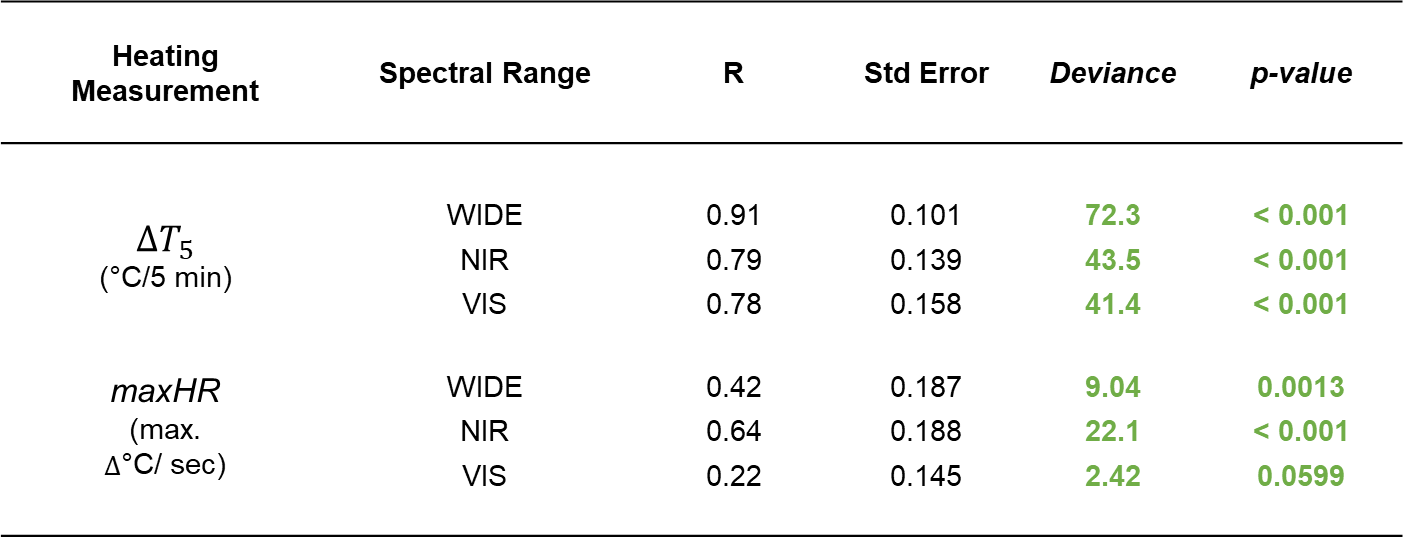
**

**
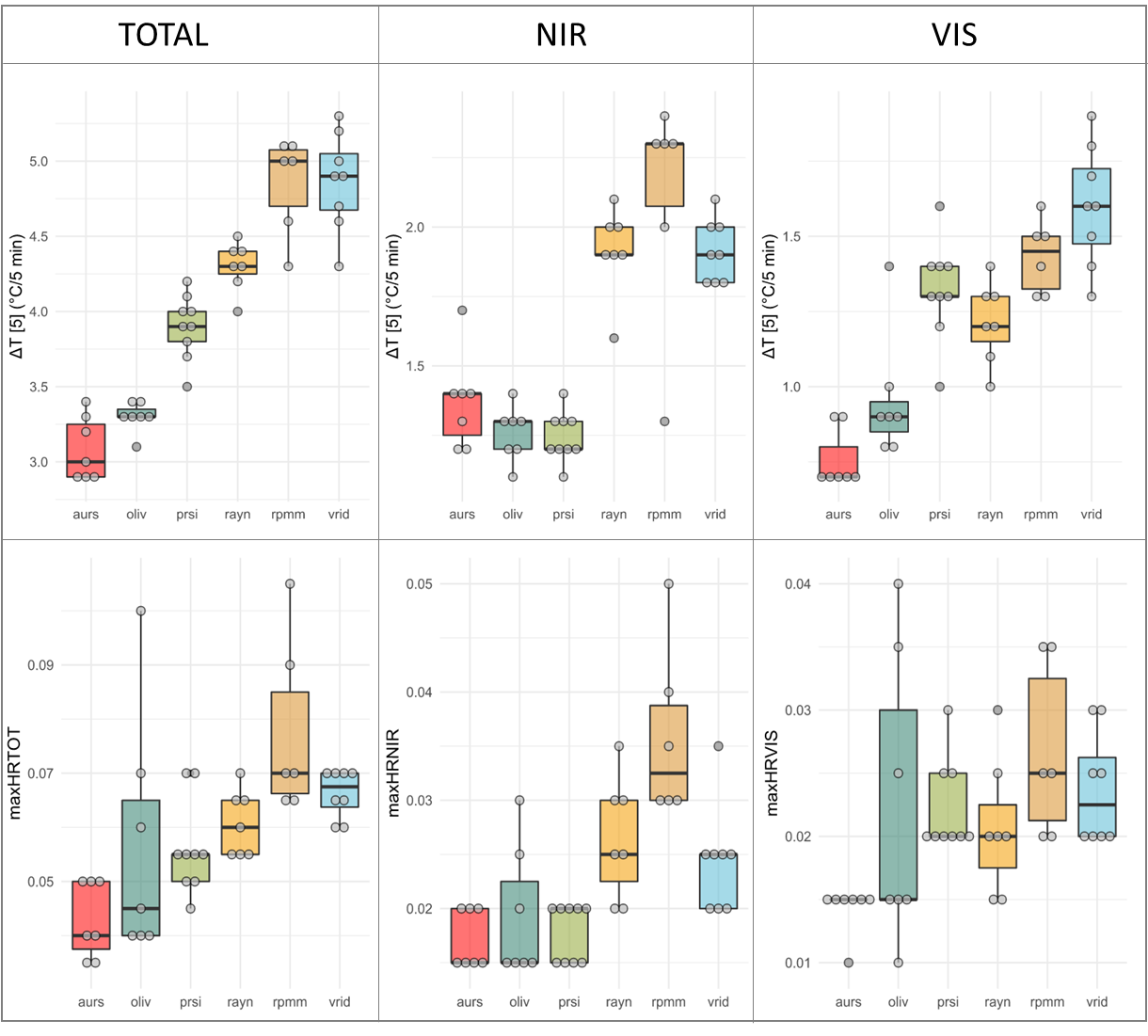
**

**Figure S3**. Boxplots showing the raw values of heating measurements for the 7 species used to test repeatability in the different spectral bands.

**Table S3**. Results of 6 models testing how heating ($\Delta T_{5}$ and maxHR) is predicted by reflectivity and size (elytra length)

**
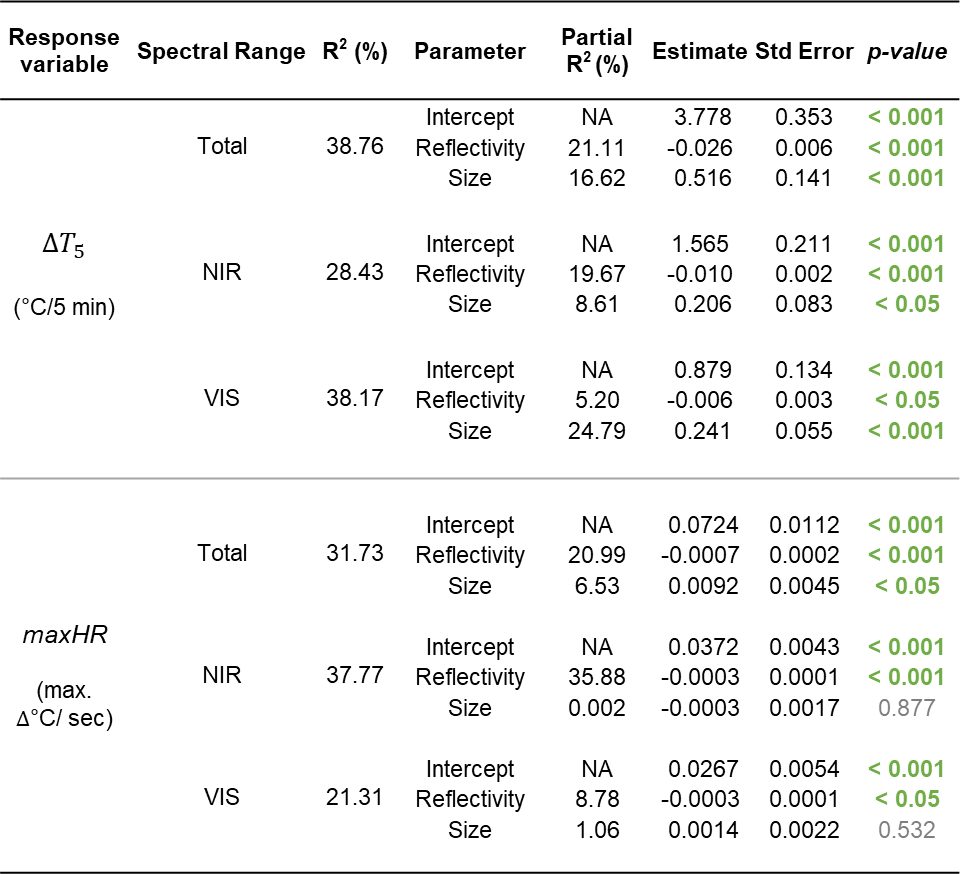
**

**Note:** The three spectral bands were TOTAL (400 – 1700 nm), NIR (700 – 1700 nm) and VIS (400 -700 nm). Units of the estimates and standard errors are specified in the response variable column. The reported estimates already account for the effect of the side of the platform in which the samples were placed. The difference between the overall R^2^ and the sum of the partial R^2^ corresponds to the variance (%) explained by the side. Its effect was significant (p < 0.05) only for $\Delta T_{5}$ and *maxHR* in VIS.

**Table S4**. Results of 6 models testing how the two response variables ($\Delta T_{5}$ and maxHR) are predicted by transmissivity and size (elytra length)

**
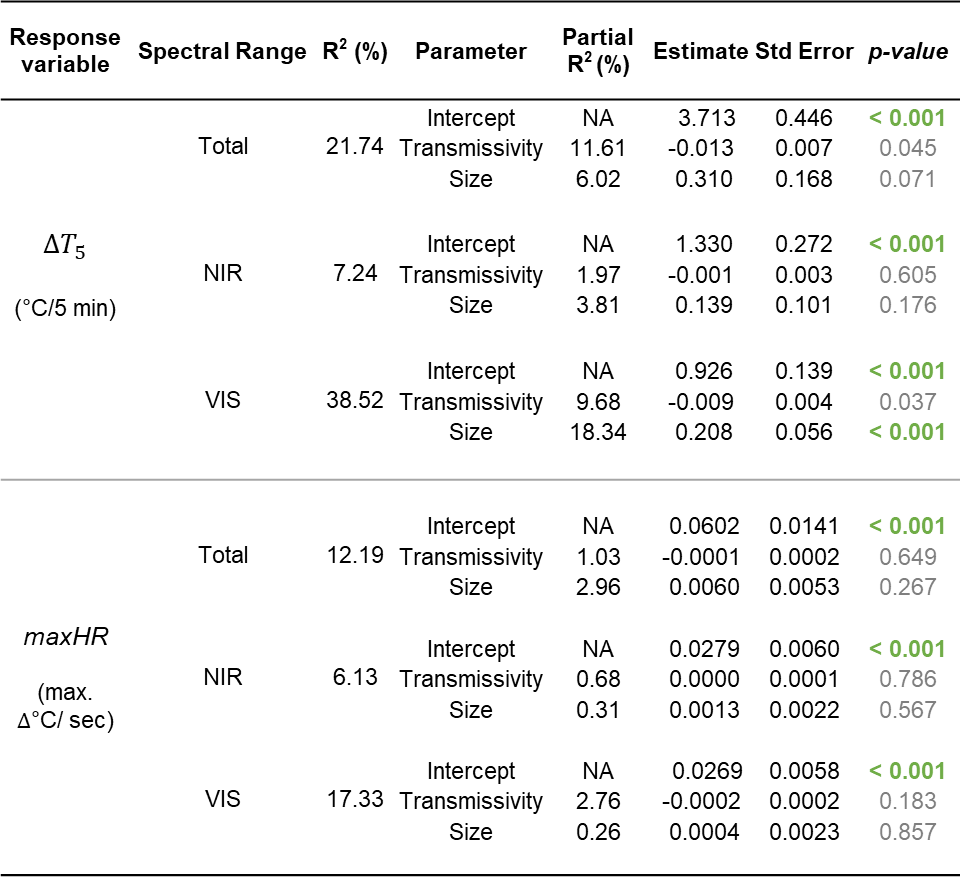
**

**Note:** The three spectral bands were TOTAL (400 – 1700 nm), NIR (700 – 1700 nm) and VIS (400 -700 nm). Units of the estimates and standard errors are specified in the response variable column. The reported estimates already account for the effect of the side of the platform in which the samples were placed. The difference between the overall R^2^ and the sum of the partial R^2^ corresponds to the variance (%) explained by the side. Its effect was significant (p < 0.05) only for $\Delta T_{5}$ and *maxHR* in TOTAL and VIS.

**S4.** *Comparison between dorsal and ventral transmittance of the elytra*

Ventral transmittance was higher by a 10.4 % in TOTAL illumination (one-sample t-test mu=0, t=2.82, df=55, p value = 0.0066) (Fig. S4B), 9.7 % in NIR (one-sample t-test mu=0, t=2.73, df=55, p value = 0.0085) and 17.97 % in VIS (one-sample t-test mu=0, t=3.14, df=55, p value = 0.0027). However, this was measured in a range of 400 to 1700 nm which does not overlap with the ranges for emissivity.


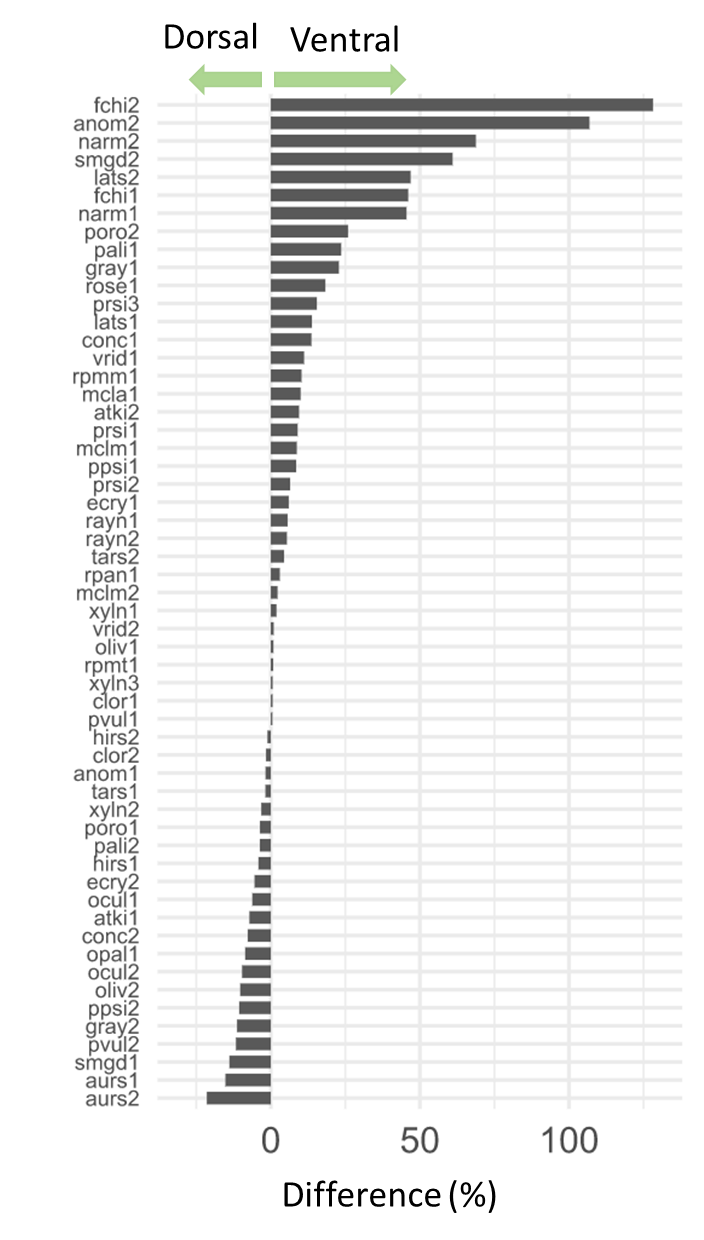


**Figure S4**. **Differences in transmittance on opposite sides of the elytra under TOTAL illumination.** The difference between dorsal and ventral transmittance, was standardized as a percentage of the dorsal transmittance. There is a subtle trend indicating higher transmittance from the ventral side of the elytra.
